# Potential Association between Breakfast Skipping and Concomitant Late-Night-Dinner Eating with Metabolic Syndrome and Proteinuria in the Japanese Population

**DOI:** 10.1155/2014/253581

**Published:** 2014-03-25

**Authors:** Ayano Kutsuma, Kei Nakajima, Kaname Suwa

**Affiliations:** ^1^Division of Clinical Nutrition, Department of Medical Dietetics, Faculty of Pharmaceutical Sciences, Josai University, 1-1 Keyakidai, Sakado, Saitama 350-0295, Japan; ^2^Saitama Health Promotion Corporation, 519 Kamiookubo, Sakura, Saitama 338-0824, Japan

## Abstract

Skipping breakfast is considered to be an unhealthy eating habit linked to predispositions to obesity and type 2 diabetes. Because eating dinner late at night can elicit subsequent breakfast skipping, we investigated if skipping breakfast concomitant with late-night-dinner eating (LNDE) was associated with metabolic syndrome (MetS) and proteinuria in the general Japanese population. We examined self-reported habitual breakfast skipping and LNDE, MetS (modified ATP-III criteria), and proteinuria in a cross-sectional study of 60,800 Japanese adults aged 20–75 years. A total of 14,068 subjects (23.1%) skipped breakfast, of whom approximately half (52.8%) skipped breakfast alone (without LNDE). The percentages of subjects who skipped breakfast showed a J-shaped relationship with body mass index (BMI). Multivariate logistic regression analysis showed that skipping breakfast concomitant with LNDE (*n* = 6,645) was significantly associated with MetS and proteinuria, even after adjusting for relevant confounders (odds ratio (95% CI), 1.17 (1.08–1.28), *P* = 0.0003, and 1.37 (1.24–1.52), *P* < 0.0001, resp.). Skipping breakfast alone and LNDE alone were not associated with MetS and proteinuria, respectively. In conclusion, habitual breakfast skipping concomitant with LNDE may represent poorer eating behavior than skipping breakfast alone, associated with MetS, asymptomatic proteinuria, obesity, and low body weight in the general Japanese population.

## 1. Introduction

Habitual skipping of breakfast is considered to be an unhealthy eating habit linked with numerous health issues, including obesity, type 2 diabetes mellitus, and metabolic syndrome (MetS) [1–6]. However, evidence for the association between skipping breakfast and these cardiometabolic abnormalities is limited, especially in Asian adults [4]. Furthermore, the mechanisms responsible for these potential associations are poorly understood. Habitual breakfast skipping is associated with various lifestyles and physical conditions, including fatigue, insomnia, lack of time for eating, smoking, infrequent exercise, alcohol drinking, full-time working, and even coronary heart disease [2, 7–12].

We focused on the possible close association between breakfast skipping and eating dinner late at night [9, 10, 13]. In Japan, dinner is usually the main meal of the day with the greatest energy content [14, 15]. However, high energy intake at night may increase the risks of obesity and type 2 diabetes [16, 17]. Late-night-dinner eating (LNDE), particularly shortly before bedtime, results in food consumption followed by a long period of inactivity and may affect subsequent physical conditions and eating behaviors. This suggests that the etiology of skipping breakfast concomitant with LNDE might differ from that of breakfast skipping without LNDE and may represent a poorer eating behavior from a cardiometabolic standpoint.

Asymptomatic proteinuria, a pivotal risk factor for cardiovascular and chronic kidney disease [18], has been observed in obese individuals [19–21]. In addition, obesity and type 2 diabetes have increased in recent decades not only in Western, but also in Asian countries [22], resulting in a pandemic of serious cardiometabolic disease [23]. In the light of these facts, we investigated if skipping breakfast, especially concomitant with LNDE, was associated with MetS, proteinuria, obesity, and other cardiometabolic risk factors in a cross-sectional study of an apparently healthy Japanese population. We also examined the associations between skipping breakfast alone (without LNDE) and LNDE alone (without skipping breakfast) and cardiometabolic conditions.

## 2. Methods

This study was based on a composite study aimed at elucidating the factors associated with cardiometabolic diseases [24]. This multicenter retrospective study began in 2011 and involved Josai University, Jichi Medical University, and the Saitama Health Promotion Corporation. The protocol conformed to the 1975 Declaration of Helsinki and was approved by the Ethics Committees of Josai University and Jichi Medical University, and the committee of the Saitama Health Promotion Corporation, which is a public interest corporation. Written informed consent was obtained from all participants.

### 2.1. Subjects

We reviewed the data for 74,805 subjects, aged 20–75 years, who underwent medical health checkups at the Saitama Health Promotion Corporation in 2008. At the checkups, all subjects were required to complete a questionnaire regarding their past medical history and lifestyle characteristics, including smoking status, alcohol intake, regular exercise, and history of diseases such as cardiovascular disease and stroke. Subjects receiving pharmacotherapy for hypertension, dyslipidemia, or diabetes were excluded because they were also likely to have received diet therapy to reduce body weight and improve eating behavior. After the additional exclusion of subjects with incomplete data, a total of 60,800 subjects (38,123 men and 22,677 women) were included in the cross-sectional study.

### 2.2. Anthropometric and Laboratory Tests

Anthropometric and laboratory tests were carried out in the morning. Body mass index (BMI) was calculated according to the following formula: weight (kg)/height (m^2^). Subjects were divided into six categories based on BMI: ≤18.9; 19.0–20.9; 21.0–22.9; 23.0–24.9; 25.0–26.9; and ≥27.0 kg/m^2^, as described elsewhere [24]. Serum parameters were measured with standard methods using Hitachi autoanalyzers (Tokyo, Japan) at the Saitama Health Promotion Corporation. HbA_1c_ (Japan Diabetes Society (JDS)) was converted to HbA_1c_ (National Glycohemoglobin Standardization Program (NGSP)) units using the officially certified formula: HbA_1c_ (NGSP) (%) = 1.02 × JDS (%) + 0.25% [25].

A series of simple questions for detecting unhealthy eating behaviors was developed by the Japanese Ministry of Health, Labour and Welfare for the nationwide Specific Health Checkups and Specific Health Guidance, started in 2008, and aimed at preventing lifestyle-related diseases [26, 27]. Habitual breakfast skipping was determined based on a positive response to the question: “Do you skip breakfast at least three times per week?” LNDE was determined based on a positive response to the question: “Do you eat dinner within two hours before bedtime at least three times per week?” In this study, LNDE did not necessarily mean that dinner was eaten particularly late (e.g., around midnight) but rather indicated that dinner was eaten shortly before the individual's bedtime, because the specific timings of meals and sleep may shift for various reasons, such as working hours and familial environments. According to the combination of answers regarding breakfast skipping and LNDE, subjects were classified into four eating-behavior groups: absence of both breakfast skipping and LNDE (normal eating behavior), LNDE alone, breakfast skipping alone, and both breakfast skipping and LNDE (skipping breakfast concomitant with LNDE).

Because of the lack of fasting plasma glucose measurement, the diagnosis of MetS was based on the modified Adult Treatment Panel III criteria [23] with the following cutoff limits: systolic blood pressure ≥ 130 mmHg or diastolic blood pressure ≥ 85 mmHg (elevated blood pressure); triglycerides ≥ 150 mg/dL; high-density lipoprotein cholesterol (HDL-C) < 40 mg/dL for men and <50 mg/dL for women (low HDL-C); HbA_1c_ ≥ 5.6% (high-normal HbA_1c_); and waist circumference ≥ 90 cm for men and ≥80 cm for women in consideration of ethnic difference. Subjects meeting three or more of these criteria were defined as having MetS. Because fasting plasma glucose data were unavailable in this study, HbA_1c_ ≥ 5.6% was used as a surrogate marker for elevated fasting plasma glucose of ≥100 mg/dL [28]. In this study, to examine the effect of severe excess body weight apart from MetS, obesity was defined as BMI ≥ 30.0 kg/m^2^, although this criterion means severe obesity in Japanese population [23].

Dipstick urinalysis was carried out using Uro-Paper III EIKEN (Eiken Chemical Co., Ltd., Tokyo, Japan) with fresh single-spot urine specimens. The results were recorded as follows: −, −/+, +1, +2+, +3, and ≥+4, which were equivalent to semiquantitative urinary protein concentrations of approximately 0, 15, 30, 100, 300, and 1,000 mg/dL, respectively. In this study, proteinuria was defined as ≥+1.

### 2.3. Statistical Analysis

Data in the tables are expressed as mean ± standard deviation or median (interquartile range). Statistical differences in categorical variables between the four eating-behavior groups and in continuous parameters between breakfast skipping concomitant with LNDE and other eating behaviors were examined by *χ* ^2^ and Bonferroni tests (post hoc analysis), respectively. Linear trends in clinical parameters across the four groups were examined by Pearson's correlation coefficients after coding the four groups as 1–4, respectively. Multivariate logistic regression models were used to examine the associations of MetS, proteinuria, and obesity with the three unhealthy eating behaviors (as independent factors), which allowed us to compare among the four groups. Tests for linear trends were calculated similarly after coding the four groups as 1–4, respectively, and the same model analysis was conducted. In the first step, the associations were not adjusted for any confounders (Model 1). Second, the associations were adjusted for age and sex (Model 2). Third, the associations were adjusted for age, sex, current smoking (versus nonsmoking), daily alcohol consumption (versus infrequent/no alcohol consumption), having regular exercise, and past history of cardiovascular disease (Model 3). Finally, the associations were examined separately according to men and women.

Subsequently, associations of each unhealthy eating behavior (as a dependent factor) with MetS, proteinuria, cardiometabolic risk factors, and the six BMI categories were also examined. In this analysis, the associations were further adjusted for MetS and proteinuria (Model 3). In contrast, the associations were adjusted for MetS components (elevated blood pressure, low HDL-C, high-normal HbA1c, and BMI categories) instead of MetS, to examine these components on the associations in detail (Model 4). Multivariate logistic regression models yielded odds ratios (ORs) and 95% confidence intervals (95% CI). Statistical analyses were performed using SPSS version 18.0 (SPSS; Chicago, IL, USA) and Statview version 5.0 (SAS Institute; Cary, NC, USA). Values of *P* < 0.05 were considered statistically significant.

## 3. Results

The clinical characteristics of the subjects stratified into the four eating-behavior groups are shown in Table 1. A total of 14,068 subjects reported breakfast skipping, among which approximately half did not report concomitant LNDE. There was a significant association between skipping breakfast and LNDE (*P* < 0.0001, *χ* ^2^-test, data not shown). Subjects who skipped breakfast, especially concomitant with LNDE, were more likely to be men, current smokers, take less regular exercise, but be younger, compared with subjects in the other eating-behavior groups. BMI, waist circumference, diastolic blood pressure, and liver enzymes were significantly higher in subjects who skipped breakfast concomitant with LNDE compared with those who skipped breakfast alone, while HDL-C and HbA_1c_ were lower than in those with LNDE alone.

**Figure 1:**
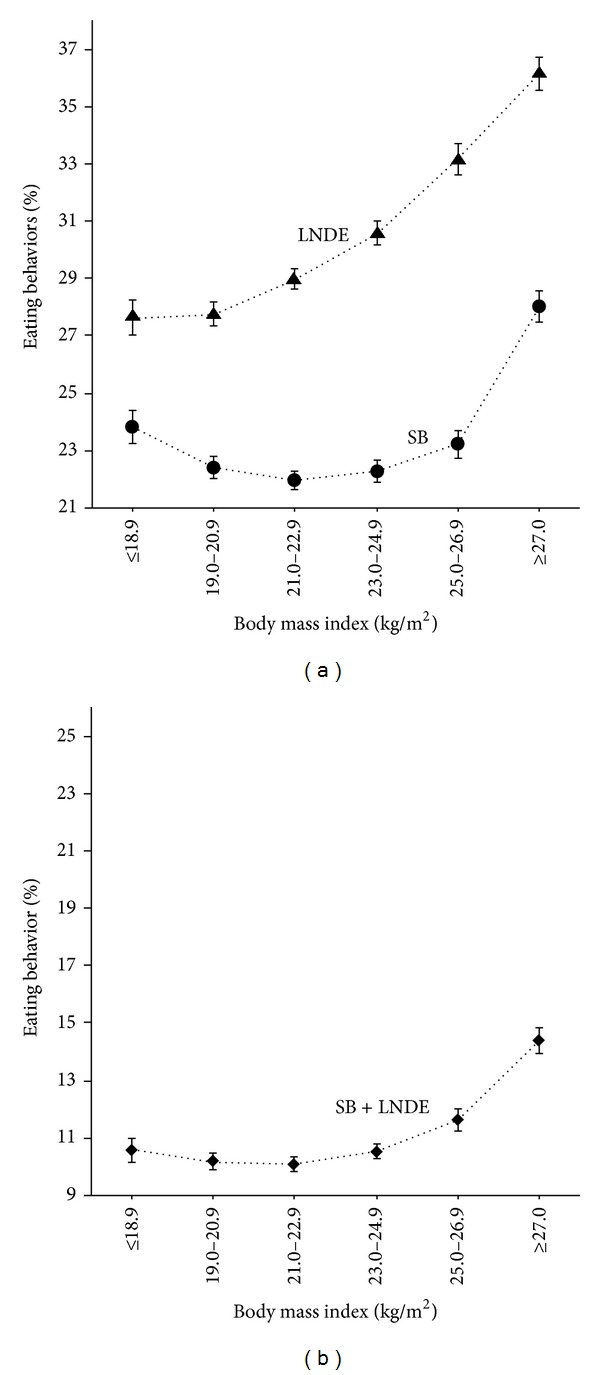
Proportions of subjects who skipped breakfast (SB) and with LNDE (a), and who skipped breakfast concomitant with LNDE (SB + LNDE), according to six body mass index (BMI) categories. The symbols in the middle of each bar represent the mean percentage of subjects with breakfast skipping, LNDE, or breakfast skipping concomitant with LNDE and was calculated as the number of subjects with these behaviors/number of subjects in each BMI group ×100, for each BMI category. The vertical bar represents the standard error (SEM) when skipping breakfast, LNDE, or skipping breakfast concomitant with LNDE was numbered as 1, and the absence of these behaviors as 0. Triangles (▲), circles (●), and diamonds (♦) represent overall breakfast skipping (SB), overall LNDE, and breakfast skipping concomitant with LNDE (SB + LNDE), respectively. The numbers of subjects in each of the six BMI categories from ≤18.9 to ≥27.0 kg/m^2^ were 5,522, 12,341, 15,708, 12,836, 7,484, and 6,909, respectively. The numbers of subjects in each category who skipped breakfast were 1,316 (9.4%), 2,765 (19.7%), 3,451 (24.5%), 2,862 (20.3%), 1,739 (12.4%), and 1,935 (13.8%); respectively, and the numbers of subjects with LNDE were 1,526 (8.3%), 3,426 (18.6%), 4,550 (24.7%), 3,925 (21.3%), 2,482 (13.5%), and 2,497 (13.6%), respectively. The numbers of subjects with SB + LNDE were 584 (8.8%), 1,256 (18.9%), 1,586 (23.9%), 1,354 (20.4%), 871 (13.1%), and 994 (15.0%), respectively (% of total SB + LNDE).

The proportions of subjects in each BMI category who skipped breakfast (including LNDE) and with LNDE (including skipping breakfast) were represented by a J-shaped curve and a positive linear relationship, respectively (Figure 1). Among the subjects who skipped breakfast, 9.4% had a low BMI of ≤18.9 kg/m^2^. The relationship between skipping breakfast concomitant with LNDE and increasing BMI showed a small J-shaped curve.

Table 2 shows the results of multivariate logistic regression analyses. Although skipping breakfast concomitant with LNDE was significantly associated with MetS, proteinuria, and obesity, even after adjusting for relevant confounding factors, skipping breakfast alone and LNDE alone were not associated with MetS and proteinuria, respectively. All *P* values for trends across the groups were significant for MetS, proteinuria, and obesity. When subjects were analyzed according to sex, no significant associations between the three unhealthy eating behaviors and MetS were observed in women.

The associations between each eating behavior and cardiometabolic conditions are shown in Table 3. Overall, both LNDE alone and skipping breakfast alone were significantly associated with current smoking and inversely associated with taking regular exercise. LNDE alone was associated with MetS but not with proteinuria, after adjusting for MetS or BMI. In contrast, skipping breakfast alone was significantly associated with elevated blood pressure and proteinuria but not with MetS, after adjusting for relevant factors. Skipping breakfast concomitant with LNDE was significantly associated with MetS and proteinuria, even after adjusting for all relevant confounders (Model 4). Unexpectedly, HDL-C was positively associated with LNDE alone and with skipping breakfast concomitant with LNDE, after adjusting for confounders. The significant associations between high-normal HbA_1c_ and LNDE alone, and skipping breakfast concomitant with LNDE, disappeared after adjusting for BMI (Model 4).

Skipping breakfast (including LNDE) and LNDE (including skipping breakfast) were significantly associated with both MetS and proteinuria, even after adjusting for the confounding factors listed in Model 3 (OR (95% CI), 1.07 (1.01–1.14), *P* = 0.04, and 1.35 (1.26–1.46), *P* < 0.0001, for skipping breakfast, and 1.15 (1.09–1.21), *P* < 0.0001, and 1.10 (1.03–1.18), *P* = 0.006, for LNDE; respectively, data is not shown in table).

We also investigated the associations between the three unhealthy eating behaviors and the six BMI categories (Model 4). LNDE alone was significantly associated with a BMI of ≥23.0 kg/m^2^. However, skipping breakfast alone was significantly associated with BMIs ≥ 27.0 kg/m^2^ and ≤18.9 kg/m^2^, relative to a BMI of 21.0–22.9 kg/m^2^. Skipping breakfast concomitant with LNDE was significantly associated with BMIs of ≤18.9 kg/m^2^ and ≥23.0 kg/m^2^. All three unhealthy eating behaviors were significantly and positively associated with continuous BMI values.

## 4. Discussion 

This study demonstrated that self-reported habitual breakfast skipping concomitant with LNDE may be associated with MetS, proteinuria, and both obesity and low body weight in the general Japanese population. However, skipping breakfast alone, as practiced by approximately half of those who skipped breakfast, was not associated with MetS. In contrast, LNDE alone, which was more common than skipping breakfast alone (19.3% of total), was not associated with proteinuria after adjusting for MetS or BMI categories, suggesting that the proteinuria detected in subjects with LNDE alone may be linked to obesity or obesity-related conditions. Overall, these results suggest that habitual breakfast skipping concomitant with LNDE may be worse than skipping breakfast alone from a cardiometabolic standpoint, and that concomitant LNDE might be an important factor to consider when exploring the etiology of breakfast skipping. Although no significant association between breakfast skipping and MetS was observed in women, the MetS criteria used or the current modification might have interfered with the outcomes, because significant associations between skipping breakfast and obesity, a fundamental factor for MetS, similar to those seen in men, were also observed in women.

Nocturnal life and irregular meal times have been shown to be associated with impaired regulation of hormones such as leptin, insulin, and glucocorticoids [9, 29]. LNDE can therefore affect cardiometabolic and endocrine functions during sleep and the following morning. Disruption of the dinner-sleep-breakfast routine by unhealthy eating behaviors around bedtime may thus increase the risks of MetS and proteinuria.

Many clinical studies have stressed the associations between habitual breakfast skipping and obesity, type 2 diabetes, and MetS [1–6]. Accordingly, this study found a positive association between skipping breakfast, regardless of LNDE, with continuous BMI variables, indicating a relationship with obesity. However, further analysis revealed that skipping breakfast was significantly associated with high as well as low BMI. In addition, the observed associations between overall breakfast skipping and obesity and obesity-related conditions, including high-normal HbA_1c_ (≥5.6%), may be due primarily to the pathogenic features of concomitant LNDE.

Unfortunately, this study did not establish the reasons for skipping breakfast, and it is therefore possible that the association between this habit and low body weight may reflect intentional breakfast skipping to lose weight. Several investigators have speculated that skipping breakfast can lead to increased hunger later in the day, which in turn promotes overeating [30], while breakfast consumption may increase satiety and reduce the hunger [31]. On the other hand, skipping breakfast represents a reduction in energy intake, which could lead to weight loss, and has been associated with decreased total energy intake in certain populations [32–34]. These results could thus explain the current observation that skipping breakfast was associated with low BMI. Similarly, we did not establish the reasons for LNDE. However, working overtime and familial factors are plausible explanations, and further consideration of these causes will give a clearer insight into the suspected cause-effect relationship between skipping breakfast and LNDE.

This study demonstrated significant positive associations between HDL-C and LNDE alone, and skipping breakfast concomitant with LNDE, after adjusting for confounders. The most likely explanation is that alcohol consumption may be greater in subjects with such eating behaviors, which may increase serum HDL-C. However, the amount of alcohol consumed was not evaluated in this study.

We previously reported a significant association between skipping breakfast and trace proteinuria (+/–) assessed by dipstick urinalysis in 2,194 apparently healthy Japanese men and women [35]; however, to the best of our knowledge, the current study is the first to demonstrate a robust association between skipping breakfast and proteinuria in a large general population. A growing body of clinical evidence has revealed that proteinuria, including albuminuria, is much more common in obese individuals than in normal-weight individuals [18–20]. However, our recent study [36] and a study by Sato et al. [37] showed that low-body-weight individuals may also be at increased risk of proteinuria, relative to normal-weight individuals. The currently observed association between proteinuria and habitual breakfast skipping is therefore not surprising. In this context, the association of breakfast skipping concomitant with LNDE with not only obesity but also low body weight may not be surprising. However, whether the association between breakfast skipping and proteinuria is mediated through abnormal body weight (i.e., obesity or low body weight) remains unknown. Because these significant associations were not appreciably altered after adjusting for BMI categories, specific independent effects of skipping breakfast might contribute to the development of proteinuria.

## 5. Limitations

Our study had several limitations. First, the questionnaire used to determine breakfast skipping and LNDE was not universally validated for the assessment of unhealthy eating behaviors. A detailed assessment of food intake using food-frequency questionnaires, for example, may be necessary to evaluate the quantity and quality of breakfast eaten and other eating behaviors. Furthermore, there is currently no worldwide standard definition for breakfast skipping [1], which may also generate bias. However, considering the large sample size in this study, a detailed assessment of food intake may not be feasible because of time and cost restrictions. Second, this study was based on cross-sectional data, which did not allow us to exclude the likelihood of inverse causality between cardiometabolic conditions and unhealthy eating behaviors. Finally, because this study included mostly nonobese individuals without pharmacotherapy for hypertension, dyslipidemia, and diabetes, the current findings may not be applicable to subjects receiving pharmacotherapy or with advanced cardiometabolic diseases, or to people with high energy intakes at lunchtime. Further prospective studies, including a more detailed assessment of eating behaviors, are needed to confirm the current observations.

## 6. Conclusions

The results of the present study suggest that habitual breakfast skipping concomitant with LNDE may represent poorer eating behavior associated with MetS, asymptomatic proteinuria, and both obesity and low body weight in asymptomatic adults.

## Figures and Tables

**Table 1:** Clinical characteristics of subjects according to four eating behavior groups.

Eating behavior groups	Normal eating behavior	LNDE alone	Skipping breakfast alone	Skipping breakfast concomitant with LNDE
*N* (% of total)	34,971 (57.5)	11,761 (19.3)	7,423 (12.2)	6,645 (10.9)
Men, *n* (%)	20,010 (57.2)	7,934 (67.5)	5,174 (69.7)	5,005 (75.3)
Age (years)	45.2 ± 12.9	42.9 ± 12.0	39.2 ± 12.0	38.5 ± 11.0^a,b^
BMI (kg/m^2^)	22.8 ± 3.2	23.2 ± 3.4	23.0 ± 3.5	23.3 ± 3.7^b^
Waist circumference (cm)	80.2 ± 9.0	81.3 ± 9.5	80.3 ± 9.6	81.4 ± 10.0^b^
Systolic blood pressure (mmHg)	121 ± 16.4	122 ± 16.2	121 ± 15.8	122 ± 15.8
Diastolic blood pressure (mmHg)	74 ± 12	75 ± 13	74 ± 12	75 ± 13^b^
Aspartate aminotransferase (IU/L)	22 (19–26)	22 (19–27)	21 (18–25)	21 (18–26)^a,b^
Alanine aminotransferase (IU/L)	18 (13–25)	19 (14–28)	18 (13–26)	19 (14–28)^b^
*γ*-glutamyltransferase (IU/L)	22 (16–36)	25 (17–43)	23 (17–37)	25 (18–43)^b^
Triglycerides (mg/dL)	90 (61–136)	91 (61–141)	90 (61–140)	91 (60–142)
HDL-cholesterol (mg/dL)	62.6 ± 15.3	62.3 ± 15.4	60.2 ± 15.1	60.7 ± 15.1^a^
HbA1c (%, NGSP)	5.5 ± 0.6	5.5 ± 0.6	5.4 ± 0.6	5.4 ± 0.6^a^
Past history of CVD, *n* (%)	450 (1.3)	159 (1.4)	85 (1.1)	67 (1.0)
Proteinuria, *n* (%)	2,009 (5.7)	741 (6.3)	623 (8.4)	568 (8.5)
Daily alcohol consumption, *n* (%)	6,887 (19.7)	3,761 (32.0)	1,491 (20.0)	1,999 (30.1)
Current smoker, *n* (%)	8,611 (24.6)	3,776 (32.1)	3,747 (50.5)	3,574 (53.8)
Having regular exercise, *n* (%)	9,028 (25.8)	2,741 (23.3)	1,495 (20.1)	1,316 (19.8)
MetS^c^, *n* (%)	4,595 (13.1)	1,610 (13.7)	900 (12.1)	849 (12.8)

*Bonferroni *P* < 0.0083, (^a^LND alone versus skipping breakfast concomitant with LNDE; ^b^skipping breakfast alone versus skipping breakfast concomitant with LNDE).

^c^MetS was determined according to the modified Adult Treatment Panel III criteria because of the lack of fasting plasma glucose measurement.

Concentrations of hepatic enzymes and triglycerides are expressed as median (interquartile range). Linear trends in clinical parameters across the four eating habit groups were examined by Pearson's correlation coefficient. Hepatic enzymes and triglycerides were log transformed before analysis. All *P* for trend values in continuous variables were <0.0001. Differences in categorical variables between the four dietary groups were examined by *χ* ^2^ tests. All *P* values were <0.0001, except for past history of CVD (*P* = 0.16) and MetS (*P* = 0.02, Cramer *V* value, 0.01).

BMI: body mass index; CVD: cardiovascular disease (including stroke); HDL: high-density lipoprotein; LNDE: late-night-dinner eating; MetS: metabolic syndrome; NGSP: National Glycohemoglobin Standardization Program.

**Table 2:** Odds ratios and 95% CI of unhealthy eating behaviors for MetS, proteinuria, and obesity.

	Normal eating behavior	LNDE alone	Skipping breakfast alone	Skipping breakfast concomitant with LNDE	*P* for trend
MetS					
Case *N*	4,595	1,610	900	849	
Model 1	1 (Ref)	1.05 (0.99–1.11)	0.91 (0.85–0.98)*	0.97 (0.90–1.05)	0.98 (0.96–1.01)
Model 2	1 (Ref)	1.14 (1.07–1.22)^††^	1.17 (1.08–1.27)^††^	1.28 (1.18–1.39)^††^	1.09 (1.06–1.11)^††^
Model 3	1 (Ref)	1.17 (1.10–1.25)^††^	1.08 (1.00–1.17)	1.21 (1.11–1.32)^††^	1.06 (1.04–1.09)^††^
Model 3					
Men	1 (Ref)	1.20 (1.12–1.30)^††^	1.08 (0.99–1.19)	1.21 (1.10–1.32)^††^	1.06 (1.03–1.09)^††^
Women	1 (Ref)	1.09 (0.96–1.23)	1.04 (0.88–1.23)	1.10 (0.91–1.34)	1.03 (0.98–1.09)
Proteinuria					
Case *N*	2,009	741	623	568	
Model 1	1 (Ref)	1.10 (1.01–1.20)*	1.50 (1.37–1.65)^††^	1.53 (1.39–1.69)^††^	1.17 (1.14–1.21)^††^
Model 2	1 (Ref)	1.08 (0.99–1.18)	1.43 (1.30–1.57)^††^	1.45 (1.31–1.60)^††^	1.15 (1.11–1.18)^††^
Model 3	1 (Ref)	1.08 (0.99–1.15)	1.37 (1.25–1.51)^††^	1.40 (1.26–1.55)^††^	1.13 (1.10–1.17)^††^
Model 3					
Men	1 (Ref)	1.09 (0.98–1.22)	1.40 (1.25–1.58)^††^	1.43 (1.27–1.61)^††^	1.14 (1.10–1.18)^††^
Women	1 (Ref)	1.04 (0.90–1.22)	1.38 (1.16–1.63)^†^	1.41 (1.16–1.71)^†^	1.13 (1.07–1.20)^††^
Obesity					
Case *N*	1,038	472	333	360	
Model 1	1 (Ref)	1.37 (1.22–1.53)^††^	1.54 (1.35–1.74)^††^	1.87 (1.66–2.12)^††^	1.23 (1.19–1.28)^††^
Model 2	1 (Ref)	1.27 (1.14–1.42)^††^	1.31 (1.16–1.49)^††^	1.57 (1.38–1.77)^††^	1.16 (1.11–1.20)^††^
Model 3	1 (Ref)	1.36 (1.22–1.53)^††^	1.28 (1.13–1.46)^†^	1.62 (1.42–1.84)^††^	1.17 (1.12–1.21)^††^
Model 3					
Men	1 (Ref)	1.30 (1.14–1.49)^†^	1.25 (1.08–1.46)^†^	1.53 (1.32–1.77)^††^	1.14 (1.10–1.18)^††^
Women	1 (Ref)	1.53 (1.25–1.88)^††^	1.32 (1.01–1.71)*	1.89 (1.45–2.46)^††^	1.13 (1.07–1.20)^††^

*P < 0.05, ^†^ *P* < 0.01, ^††^ *P* < 0.0001.

Model 1: Unadjusted.

Model 2: Adjusted for age and sex.

Model 3: Adjusted for age, sex, current smoking (versus nonsmoking), daily alcohol consumption (versus infrequent/no alcohol consumption), having regular exercise, and past history of cardiovascular disease.

Obesity was defined as body mass index ≥30.0 kg/m^2^.

**Table 3:** Odds ratios and 95% CIs of unhealthy eating behaviors for cardiometabolic conditions.

	Model 1	Model 2	Model 3	Model 4
LNDE alone				
(total *n* = 46,732)^a^				
Current smoking	1.46 (1.39–1.52)^††^	1.16 (1.10–1.21)^††^	1.15 (1.09–1.21)^††^	1.16 (1.10–1.22)^††^
Daily alcohol drinking	2.27 (2.15–2.39)^††^	2.20 (2.08–2.32)^††^	2.23 (2.10–2.36)^††^	2.23 (2.10–2.36)^††^
Having regular exercise	0.87 (0.83–0.91)^††^	0.84 (0.80–0.88)^††^	0.85 (0.81–0.89)^††^	0.85 (0.80–0.89)^††^
Elevated blood pressure	1.11 (1.06–1.16)^††^	1.07 (1.02–1.12)^†^		1.01 (0.96–1.06)
Low HDL-C	0.83 (0.77–0.91)^††^	0.97 (0.89–1.06)		0.89 (0.81–0.97)*
High–normal HbA1c	0.92 (0.87–0.97)^†^	1.07 (1.01–1.13)*		1.02 (0.97–1.08)
Proteinuria	1.11 (1.02–1.21)*	1.09 (1.00–1.19)*	1.08 (0.99–1.18)	1.06 (0.97–1.16)
Continuous BMI		1.03 (1.03–1.04)^††^		
MetS		1.15 (1.08–1.23)^††^	1.15 (1.08–1.23)^††^	
Six BMI categories (*N*)				
≤18.9 kg/m^2^ (4,206)				0.97 (0.89–1.06)
19.0–20.9 kg/m^2^ (9,576)				0.95 (0.89–1.02)
21.0–22.9 kg/m^2^ (12,257)				1 (reference)
23.0–24.9 kg/m^2^ (9,974)				1.08 (1.01–1.15)*
25.0–26.9 kg/m^2^ (5,745)				1.21 (1.12–1.30)^††^
≥27.0 kg/m^2^ (4,974)				1.34 (1.24–1.45)^††^
Skipping breakfast alone				
(total *n* = 42,394)^b^				
Current smoking	3.13 (2.98–3.29)^††^	2.88 (2.72–3.04)^††^	2.84 (2.68–3.01)^††^	2.85 (2.69–3.02)^††^
Daily alcohol drinking	1.17 (1.10–1.26)^††^	1.03 (0.95–1.11)	1.03 (0.96–1.11)	1.03 (0.95–1.11)
Having regular exercise	0.73 (0.69–0.78)^††^	0.73 (0.68–0.78)^††^	0.72 (0.68–0.77)^††^	0.73 (0.68–0.78)^††^
Elevated blood pressure	0.96 (0.91–1.01)	1.12 (1.06–1.19)^†^		1.10 (1.03–1.17)^†^
Low HDL-C	1.05 (0.95–1.15)	1.02 (0.92–1.12)		0.98 (0.89–1.09)
High-normal HbA1c	0.74 (0.69–0.79)^ ††^	0.99 (0.92–1.07)		0.97 (0.90–1.04)
Proteinuria	1.50 (1.37–1.64)^ ††^	1.35 (1.23–1.49)^††^	1.35 (1.22–1.49)^††^	1.33 (1.21–1.47)^††^
Continuous BMI		1.01 (1.00–1.02)*		
MetS		1.07 (0.99–1.16)	1.06 (0.97–1.15)	
Six BMI categories (*N*)				
≤18.9 kg/m^2^ (3,996)				1.15 (1.04–1.27)^†^
19.0–20.9 kg/m^2^ (8,915)				1.05 (0.97–1.14)
21.0–22.9 kg/m^2^ (11,158)				1 (reference)
23.0–24.9 kg/m^2^ (8,911)				1.03 (0.95–1.11)
25.0–26.9 kg/m^2^ (5,002)				1.00 (0.91–1.10)
≥27.0 kg/m^2^ (4,412)				1.18 (1.07–1.30)^†^
Skipping breakfast concomitant with LNDE				
(total *n* = 41,616)^c^				
Current smoking	3.56 (3.38–3.76)^††^	2.79 (2.63–2.96)^††^	2.76 (2.60–2.92)^††^	2.78 (2.62–2.96)^††^
Daily alcohol drinking	2.12 (1.98–2.27)^††^	1.90 (1.77–2.05)^††^	1.93 (1.78–2.08)^††^	1.92 (1.78–2.08)^††^
Having regular exercise	0.71 (0.67–0.76)^††^	0.69 (0.64–0.74)^††^	0.69 (0.64–0.74)^††^	0.69 (0.65–0.74)^††^
Elevated blood pressure	1.05 (1.00–1.11)	1.16 (1.09–1.23)^††^		1.07 (1.00–1.14)
Low HDL-C	0.86 (0.77–0.96)^†^	0.92 (0.82–1.03)		0.84 (0.75–0.95)^†^
High-normal HbA1c	0.76 (0.71–0.82)^††^	1.10 (1.02–1.19)*		1.04 (0.96–1.13)
Proteinuria	1.53 (1.39–1.69)^††^	1.39 (1.25–1.54)^††^	1.37 (1.24–1.52)^††^	1.34 (1.21–1.48)^††^
Continuous BMI		1.04 (1.03-1.04)^††^		
MetS		1.19 (1.09–1.29)^††^	1.17 (1.08–1.28)^†^	
Six BMI categories (*N*)				
≤18.9 kg/m^2^ (3,848)				1.16 (1.04–1.29)^†^
19.0–20.9 kg/m^2^ (8,662)				1.06 (0.97–1.15)
21.0–22.9 kg/m^2^ (10,879)				1 (reference)
23.0–24.9 kg/m^2^ (8,757)				1.10 (1.01–1.19)*
25.0–26.9 kg/m^2^ (5,005)				1.20 (1.09–1.32)^†^
≥27.0 kg/m^2^ (4,465)				1.54 (1.40–1.70)^††^

The number of total subjects in each analysis comprises that of normal eating behavior (*n* =  34,971) plus that of LNDE alone (*n* =  11,761)^a^, skipping breakfast alone (*n* =  7,423)^b^, or skipping breakfast concomitant with LNDE (*n* =  6,645)^c^.

*P < 0.05, ^†^ *P* < 0.01, ^††^ *P* < 0.0001.

The number of subjects and those with confounding factors in each unhealthy eating behavior group is the same as that in Table 1.

Model 1: Unadjusted.

Model 2: Adjusted for age, sex, current smoking (versus noncurrent smoking), daily alcohol consumption (versus infrequent/no alcohol consumption), having regular exercise (versus nonregular exercise), and past history of cardiovascular disease (versus nonpast history).

Model 3: Adjusted as for Model 2 plus MetS and proteinuria.

Model 4: Adjusted as for Model 2 plus elevated blood pressure, low HDL-C, high-normal HbA1c, BMI categories, and proteinuria.

## Abbreviations

MetS: Metabolic syndrome
LNDE: Late-night-dinner eating
BMI: Body mass index
NGSP: National Glycohemoglobin Standardization Program
JDS: Japan Diabetes Society
HDL-C: High-density lipoprotein cholesterol
ORs: Odds ratios
95% CI: 95% confidential interval.

## References

[1] Timlin MT, Pereira MA (2007). Breakfast frequency and quality in the etiology of adult obesity and chronic diseases. *Nutrition Reviews*.

[2] Huang C-J, Hu H-T, Fan Y-C, Liao Y-M, Tsai P-S (2010). Associations of breakfast skipping with obesity and health-related quality of life: evidence from a national survey in Taiwan. *International Journal of Obesity*.

[3] Pereira MA, Erickson E, McKee P (2011). Breakfast frequency and quality may affect glycemia and appetite in adults and children. *Journal of Nutrition*.

[4] Horikawa C, Kodama S, Yachi Y (2011). Skipping breakfast and prevalence of overweight and obesity in Asian and Pacific regions: a meta-analysis. *Preventive Medicine*.

[5] Odegaard AO, Jacobs DR, Steffen LM, van Horn L, Ludwig DS, Pereira MA (2013). Breakfast frequency and development of metabolic risk. *Diabetes Care*.

[6] Soga Y, Shirai C, Ijichi A (2013). Association between daily lifestyle and the risk of metabolic syndrome among young adults in Japan: an analysis of Kobe city young adult health examination data. *Nihon Koshu Eisei Zasshi*.

[7] Tanaka M, Mizuno K, Fukuda S, Shigihara Y, Watanabe Y (2008). Relationships between dietary habits and the prevalence of fatigue in medical students. *Nutrition*.

[8] Kaneita Y, Ohida T, Osaki Y (2006). Insomnia among Japanese adolescents: a nationwide representative survey. *Sleep*.

[9] Qin L-Q, Li J, Wang Y, Wang J, Xu J-Y, Kaneko T (2003). The effects of nocturnal life on endocrine circadian patterns in healthy adults. *Life Sciences*.

[10] Li Y, Nemoto T, Tobimatsu S (2011). Relationship between skipping breakfast and impaired fasting glucose along with cardiovascular and pre-diabetes condition risk factors in apparently healthy subjects. *Endocrinology Studies*.

[11] Keski-Rahkonen A, Kaprio J, Rissanen A, Virkkunen M, Rose RJ (2003). Breakfast skipping and health-compromising behaviors in adolescents and adults. *European Journal of Clinical Nutrition*.

[12] Cahill LE, Chiuve SE, Mekary RA (2013). Prospective study of breakfast eating and incident coronary heart disease in a cohort of male US health professionals. *Circulation*.

[13] http://www8.cao.go.jp/syokuiku/more/research/h24/3-2.html.

[14] Takahashi T, Tomizawa M, Ito K, Morino M, Uenishi K, Ishida H (2008). The distribution of energy intake in a day for married male workers living in metropolitan areas. *Japan Society of Nutrition and Food Science*.

[15] Watanabe M, Yamaoka K, Yokotsuka M, Adachi M, Tango T (2011). Validity and reproducibility of the FFQ (FFQW82) for dietary assessment in female adolescents. *Public Health Nutrition*.

[16] de Castro JM (2009). When, how much and what foods are eaten are related to total daily food intake. *British Journal of Nutrition*.

[17] Morgan LM, Shi JW, Hampton SM, Frost G (2012). Effect of meal timing and glycaemic index on glucose control and insulin secretion in healthy volunteers. *British Journal of Nutrition*.

[18] Levey AS, Coresh J, Balk E (2003). National kidney foundation practice guidelines for chronic kidney disease: evaluation, classification, and stratification. *Annals of Internal Medicine*.

[19] Praga M, Morales E (2006). Obesity, proteinuria and progression of renal failure. *Current Opinion in Nephrology and Hypertension*.

[20] Shen W-W, Chen H-M, Chen H, Xu F, Li L-S, Liu Z-H (2010). Obesity-related glomerulopathy: body mass index and proteinuria. *Clinical Journal of the American Society of Nephrology*.

[21] Eknoyan G (2011). Obesity and chronic kidney disease. *Nefrologia*.

[22] Yoon K-H, Lee J-H, Kim J-W (2006). Epidemic obesity and type 2 diabetes in Asia. *The Lancet*.

[23] Grundy SM, Cleeman JI, Daniels SR (2005). Diagnosis and management of the metabolic syndrome: an American Heart Association/National Heart, Lung, and Blood Institute scientific statement. *Circulation*.

[24] Muneyuki T, Suwa K, Oshida H (2013). Design of the saitama cardiometabolic disease and organ impairment study (SCDOIS): a multidisciplinary observational epidemiological study. *Open Journal of Endocrine and Metabolic Diseases*.

[25] Kashiwagi A, Kasuga M, Araki E (2012). International clinical harmonization of glycated hemoglobin in Japan: from Japan Diabetes Society to National Glycohemoglobin Standardization Program values. *Journal of Diabetes Investigation*.

[26] Ministry of Health http://www.mhlw.go.jp/bunya/shakaihosho/iryouseido01/info02a.html.

[27] Soga Y, Shirai C, Ijichi A (2013). Association between daily lifestyle and the risk of metabolic syndrome among young adults in Japan: an analysis of Kobe city young adult health examination data. *Nihon Koshu Eisei Zasshi*.

[28] Seino Y, Nanjo K, Tajima N (2010). Report of the committee on the classification and diagnostic criteria of diabetes mellitus. *Diabetology International*.

[29] Elimam A, Marcus C (2002). Meal timing, fasting and glucocorticoids interplay in serum leptin concentrations and diurnal profile. *European Journal of Endocrinology*.

[30] Kral TV, Whiteford LM, Heo M, Faith MS (2011). Effects of eating breakfast compared with skipping breakfast on ratings of appetite and intake at subsequent meals in 8- to 10-y-old children. *American Journal of Clinical Nutrition*.

[31] Leidy HJ, Racki EM (2010). The addition of a protein-rich breakfast and its effects on acute appetite control and food intake in “breakfast-skipping” adolescents. *International Journal of Obesity*.

[32] Cho S, Dietrich M, Brown CJ, Clark CA, Block G (2003). The effect of breakfast type on total daily energy intake and body mass index: results from the Third National Health and Nutrition Examination Survey (NHANES III). *Journal of the American College of Nutrition*.

[33] Min C, Noh H, Kang Y-S (2011). Skipping breakfast is associated with diet quality and metabolic syndrome risk factors of adults. *Nutrition Research and Practice*.

[34] Levitsky DA, Pacanowski CR (2013). Effect of skipping breakfast on subsequent energy intake. *Physiology & Behavior*.

[35] Nemoto T, Yamaoka H, Sato C (2011). Association between trace proteinuria and elevated waist circumference: results of cross-sectional study of asymptomatic adults. *Journal of Japanese Society of Clinical Nutrition*.

[36] Muneyuki T, Sugawara H, Suwa K (2013). A community-based cross-sectional and longitudinal study uncovered asymptomatic proteinuria in Japanese adults with low body weight. *Kidney International*.

[37] Sato Y, Fujimoto S, Konta T (2014). U-shaped association between body mass index and proteinuria in a large Japanese general population sample. *Clinical and Experimental Nephrology*.

